# OP09 Investigating The Nature And Scope Of Innovative Payment And Pricing Schemes For Health Technologies

**DOI:** 10.1017/S0266462324000734

**Published:** 2025-01-07

**Authors:** Vittoria Ardito, Ludovico Cavallaro, Michael Drummond, Oriana Ciani

## Abstract

**Introduction:**

Innovative pricing and payment schemes have been proposed to address the affordability issues raised by new health technologies or the uncertainty about their long-term safety and effectiveness. As part of the Horizon Europe project HI-PRIX, we investigated the nature, scope, and impact of these arrangements.

**Methods:**

We undertook a PRISMA-ScR-compliant review in PubMed, Web of Science, and Scopus, from 2010 to 2023. We also searched health technology assessment (HTA) agency websites. The search strategy was structured around two blocks: “pricing/payment schemes,” and “innovativeness.” Studies illustrating pricing/payment schemes with sufficient level of details to explain their functioning were selected, also through a nested evaluation of an artificial-intelligence-powered tool for systematic reviews. These schemes were classified according to several criteria, such as their purpose, nature, governance, product category, data collection needs, foreseen distribution of risk, and implementation challenges. The study protocol was published in PROSPERO (CRD42023444824).

**Results:**

“Innovative payment and pricing schemes” were defined as arrangements that go beyond price per unit of the technology, simple price/volume agreements, or expenditure caps. Seventy innovative schemes were identified, of which 25 were only illustrated theoretically, while 45 have been implemented in practice. So far, 170 real cases of implementation have been identified. The schemes target pharmaceuticals, vaccines, and/or medical devices. Whether designed to incorporate unique features of a given technology, or to address specific challenges, the schemes can be classified by different value drivers, including type of technology, therapeutic indication, or timeline of the agreement.

**Conclusions:**

Available pricing and payment schemes have the potential to offer a comprehensive toolkit to policymakers facing reimbursement and access decisions, highlighting that it is not the scheme per se that is innovative, rather its application/use in a given context or for a given challenge. The catalog populates the Pay for Innovation Observatory, a publicly available repository.

